# Identification of core genes in ovarian cancer by an integrative meta-analysis

**DOI:** 10.1186/s13048-018-0467-z

**Published:** 2018-11-19

**Authors:** Wenyu Li, Zheran Liu, Bowen Liang, Siyang Chen, Xinping Zhang, Xiaoqin Tong, Weiming Lou, Lulu Le, Xiaoli Tang, Fen Fu

**Affiliations:** 1 The Second Affiliated Hospital of Nanchang University, Nanchang, Jiangxi 330031 People’s Republic of China; 2 Queen Mary School, Medical College of Nanchang University, Nanchang, Jiangxi 330031 People’s Republic of China; 3 School of Public Health, Nanchang University, Nanchang, Jiangxi 330031 People’s Republic of China; 4 School of Basic Medical Science, Nanchang University, Nanchang, Jiangxi 330031 People’s Republic of China

**Keywords:** Ovarian cancer, Meta-analysis, Differentially expressed genes

## Abstract

**Background:**

Epithelial ovarian cancer is one of the most ﻿severe public health threats in women. Since it is still challenging to screen in early stages, identification of core genes that play an essential role in epithelial ovarian cancer initiation and progression is of vital importance.

**Results:**

Seven gene expression datasets (GSE6008, GSE18520, GSE26712, GSE27651, GSE29450, GSE36668, and GSE52037) containing 396 ovarian cancer samples and 54 healthy control samples were analyzed to identify the significant differentially expressed genes (DEGs). We identified 563 DEGs, including 245 upregulated and 318 downregulated genes. Enrichment analysis based on the gene ontology (GO) functions and Kyoto Encyclopedia of Genes and Genomes (KEGG) pathways showed that the upregulated genes were significantly enriched in cell division, cell cycle, tight junction, and oocyte meiosis, while the downregulated genes were associated with response to endogenous stimuli, complement and coagulation cascades, the cGMP-PKG signaling pathway, and serotonergic synapse. Two significant modules were identified from a protein-protein interaction network by using the Molecular Complex Detection (MCODE) software. Moreover, 12 hub genes with degree centrality more than 29 were selected from the protein-protein interaction network, and module analysis illustrated that these 12 hub genes belong to module 1. Furthermore, Kaplan-Meier analysis for overall survival indicated that 9 of these hub genes was correlated with poor prognosis of epithelial ovarian cancer patients.

**Conclusion:**

The present study systematically validates the results of previous studies and fills the gap regarding a large-scale meta-analysis in the field of epithelial ovarian cancer. Furthermore, hub genes that could be used as a novel biomarkers to facilitate early diagnosis and therapeutic approaches are evaluated, providing compelling evidence for future genomic-based individualized treatment of epithelial ovarian cancer.

**Electronic supplementary material:**

The online version of this article (10.1186/s13048-018-0467-z) contains supplementary material, which is available to authorized users.

## Introduction

Ovarian cancer is the most lethal gynecological cancer in the world and a general term which contains some cancers derived from various tissues [1]. Epithelial ovarian cancer is the most common and representative histological types in primary ovarian cancer and is the primary cause of deaths in female cancer patients in North America and over 100,000 deaths every year worldwide [2]. High-grade serous carcinoma (HGSC) is the most lethal subtype in the epithelial ovarian cancer, and most of them are diagnosed in an advanced stage [3]. The standard treatment for ovarian cancer is maximal cytoreductive surgery and platinum-based chemotherapy [4]. Although ovarian cancer actively responds to the initial anticancer therapy, nearly 75% of patients may relapse within two years and cannot be treated with the available chemotherapy drugs [5, 6]. Meanwhile, metastasis within the peritoneal cavity and resistance to chemotherapy are the leading causes of the high mortality rate associated with ovarian cancer, because this cancer is often diagnosed in late clinical stages as what has been mentioned above [5]. Thus, identifying new targets for treatment and seeking effective chemotherapy drugs are crucial for overcoming drug resistance in advanced ovarian cancer [7].

Thus far, many genetic factors, such as *BRCA1*, *BRCA2*, *P53 (TP53)*, *KRAS*, *PIK3CA*, *CTNNB1*, and *PTEN*, have been correlated with ovarian cancer [8]. In recent years, many studies have shown promise for gene-targeted therapies in ovarian cancer [9–11]. The poly-ADP-ribose polymerase (PARP) inhibitor olaparib, a targeted therapeutic drug approved by the Food and Drug Administration, is used to treat ovarian cancer patients with BRCA1 and BRCA2 mutations. Olaparib has also been used as maintenance therapy for patients with platinum-sensitive recurrent BRCA-mutated ovarian cancer [12]. Therefore, gene-targeted therapies provide a new possibility for the personalized treatment of ovarian cancer patients.

However, the lack of large-scale studies for the identification of differentially expressed genes (DEGs) in ovarian cancer limits the reliability of previous results and makes it difficult to screen potential targets. DNA microarray analysis is a systematic and global approach to analyze genomic expression profiles and physiological mechanisms in diseases [13, 14]. High-throughput microarray experiments have been used to analyze gene expression patterns and identify potential target genes in ovarian cancer [15].

To fill the gap regarding the identification of DEGs in ovarian cancer, we performed this meta-analysis to identify DEGs between ovarian cancer and healthy control tissues and aimed to provide a powerful tool to investigate microarray datasets by integrating data from multiple studies. An important advantage of this large-scale analysis lies in the reduction of discrepancies among different study conditions; additionally, this analysis combines the results from previous studies to assess an existing problem from a novel perspective [16]. it is worth noting that although ovarian cancer is known as a series of different molecular and histological diseases [17], we aim to uncover those common genes across different molecular subtypes of epithelial ovarian cancer. Genes discovered in meta-analyses generally overlap with genes identified in various studies, indicating increased reliability [18]. The present meta-analysis aimed to identify DEGs between ovarian tissues and control tissues. In addition, we attempted to identify potential core genes associated with epithelial ovarian cancer and to investigate some possible related mechanisms.

## Materials and methods

### Selection of microarray datasets for the meta-analysis

According to the Preferred Reporting Items for Systematic Reviews and Meta-Analyses (PRISMA) guidelines published in 2009, we performed a comprehensive search of Gene Expression Omnibus (GEO) databases from the National Center for Biotechnology Information (NCBI, http://www.ncbi.nlm.nih.gov/geo/). The keywords “ovarian neoplasms” and “ovarian cancer” were used in our search. The datasets were required to meet the following criteria: (1) the samples had to be from the Affymetrix Human Genome U133A Array platform or Affymetrix Human Genome U133 Plus 2.0 Array platform; (2) the study organisms had to be *Homo sapiens*; (3) the datasets had to contain ovarian cancer and normal ovarian tissue samples; and (4) the number of normal ovarian tissue samples had to be greater than three. Studies were excluded if (1) they were cell line studies; (2) they involved dual-channel arrays; (3) they did not have literature traceability; (4) they were DNA methylation studies; (5) they were miRNA-based studies; and (6) they lacked cases and controls. The data from the original studies were selected by two independent analysts. Any disagreement between the two analysts was solved by consultation with a third analyst.

### Meta-analysis of multiple microarray datasets

From the GEO database, we downloaded Ovarian files (.CEL) of microarray datasets that met the inclusion criteria. A total of 13,294 common genes were obtained by integrating the genes from seven datasets using the R statistical software (http://www.r-project.org/). Then, we performed a meta-analysis of gene expression profiles of the 13,294 common genes according to combined *p*-values and Z scores using R statistical software. Combined p-values (pval_test) included the test-statistic and p-value. The meta-analysis of common genes was conducted by the MAMA, mataMA, affyPLM, CLL and RankProd packages. In performing two meta-analysis with R statistical software, combined p-values method (where the threshold was an absolute value more than 5) and Z-scored meta-analysis (where the threshold was an absolute value more than 7) were used as the cutoff criteria, and a list of DEGs (up- or downregulated) was identified.

### GO annotations and KEGG pathway enrichment analysis

Identifying the biological characteristics of DEGs is vital. Based on the results of the meta-analysis, the most significant DEGs were evaluated by enrichment analyses. Then, gene ontology (GO) annotations and Kyoto Encyclopedia of Genes and Genomes (KEGG) pathway enrichment analyses were conducted to identify the most significant DEGs using the WEB-based GEne SeT AnaLysis Toolkit (http://www.webgestalt.org/option.php) with a significance threshold of false-discovery rate (FDR) less than 0.1.

### PPI network construction

The Search Tool for the Retrieval of Interacting Genes (STRING) database (http://string-db.org) displays information on protein-protein interactions (PPIs) [19]. We charted a PPI network of DEGs using STRING with confidence score more than 0.7 as the significance cutoff criterion to acquire an in-depth understanding and predict the cellular functions and biological behaviors of the identified DEGs. Further, PPI networks were visualized utilizing the Cytoscape software [19].

### Selection of hub genes and modules

CentiScaPe 2.1 was used to calculate the degree, closeness, and betweenness of the PPI network. The degree of a node is the average number of edges (interactions) incident on the node [20]. According to the degree of a node, we identified the hub genes. The Molecular Complex Detection (MCODE) software was employed to select the most important clustering modules of PPI networks in Cytoscape with degree cutoff = 2, node score cutoff = 0.2, k-core = 2, and max. Depth = 100. Furthermore, KEGG pathway enrichment analysis was conducted for DEGs in every module using the WEB-based GEne SeT AnaLysis Toolkit with a significance threshold of FDR less than 0.1.

### Survival analysis using hub genes

The Kaplan-Meier plotter (KM plotter, http://kmplot.com/analysis/) was used to display the relevance of the identified genes regarding patient survival using 1816 ovarian cancer samples. Gene expression data and relapse-free and overall survival (OS) information were downloaded from the GEO (Affymetrix microarrays only), the European Genome-phenome Archive (EGA) and the Cancer Genome Atlas (TCGA) databases. Hazard ratios (HRs) with 95% confidence intervals and log-rank *p*-value were calculated and displayed on the plot.

## Results

### Identification of upregulated or downregulated DEGs through the meta-analysis

**Fig. 1:**
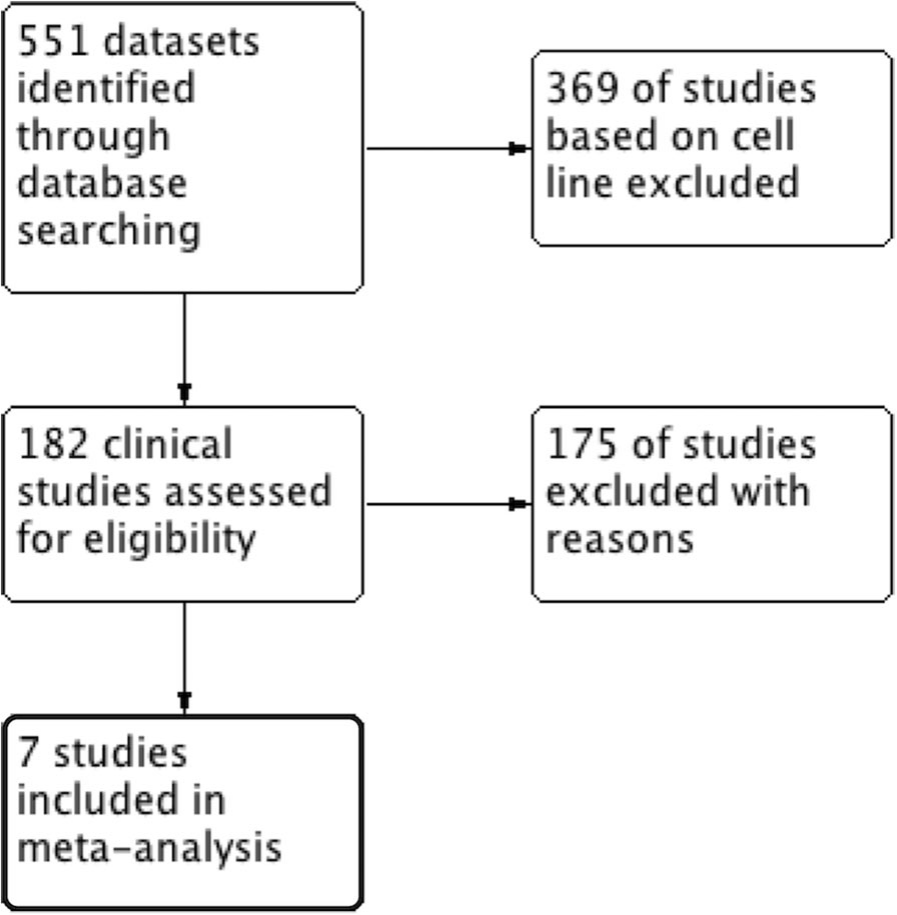
Selection procession of microarray datasets for meta-analysis

According to the inclusion criteria, the following seven GEO datasets from the NCBI were obtained: GSE6008, GSE18520, GSE26712, GSE27651, GSE29450, GSE36668, and GSE52037 (see “Materials and Methods”, Fig. 1). A total of 396 ovarian cancer samples and 54 normal ovarian tissue samples were analyzed. The GEO Platform Files (GPLs) from the seven datasets were obtained using Affymetrix gene chips (Table 1).

**Table 1:** Characteristic of individual studies retrieved from Gene Expression Omnibus for meta-analysis

Dataset	Samples	Case/Control	Country	PMID	Platforms	Gene#	Gene chip
GSE6008	103	99/4	USA	16,452,189/ 19,843,521/ 17,418,409/ 27538791	GPL96	13,909	Affymetrix Human Genome U133A Array
GSE18520	63	53/10	USA	19,962,670	GPL570	22,838	Affymetrix Human Genome U133 Plus 2.0 Array
GSE26712	195	185/10	USA	18,593,951/25944803	GPL96	13,909	Affymetrix Human Genome U133A Array
GSE27651	41	35/6	USA	21,451,362	GPL570	22,838	Affymetrix Human Genome U133 Plus 2.0 Array
GSE29450	20	10/10	USA	21,754,983	GPL570	22,838	Affymetrix Human Genome U133 Plus 2.0 Array
GSE36668	8	4/4	Norway	23,029,477	GPL570	22,838	Affymetrix Human Genome U133 Plus 2.0 Array
GSE52037	20	10/10	USA	24,666,724	GPL570	19,257	Affymetrix Human Genome U133 Plus 2.0 Array

**Fig. 2:**
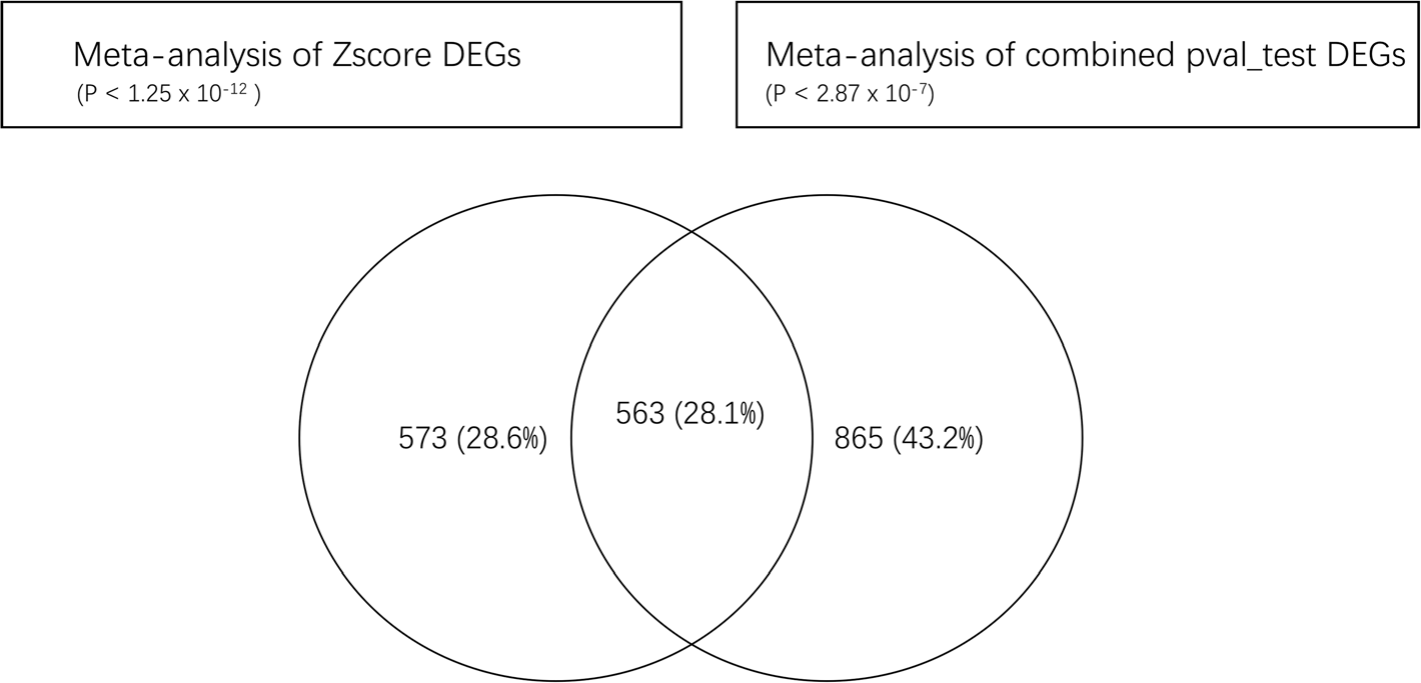
The 563 overlapping DEGs based on |pval_test| < 5 and |Z| > 7 were detected using Venny 2.1.0

We identified common genes across all datasets and performed a meta-analysis of multiple gene expression profiles using two platforms according to combined *p*-values and Z scores. According to the combined *p*-values (the threshold was 5) and Z scores (the limit was 7), 563 DEGs including 245 upregulated and 318 downregulated genes were identified (Additional file 1: Table S1 and Additional file 2: Table S2). The overlapping DEGs based on the combined *p*-values and Z scores are shown in Fig. 2.

### GO term and KEGG pathway enrichment analyses

To further investigate the functions of the DEGs, we separately classified the upregulated and downregulated genes into functional GO and KEGG categories and then performed pathway enrichment analyses with a significance threshold less than 0.05. The top five terms enriched in each category were selected according to the *p*-value.

For biological processes, GO analysis results showed that the upregulated DEGs were separately enriched in ‘cell division’ (GO:0051301), ‘cell-cell junction’ (GO:0005911) and ‘enzyme binding’ (GO:0019899), whereas and the downregulated DEGs were enriched in ‘response to endogenous stimulus’ (GO:0009719), ‘extracellular space’ (GO:0005615) and ‘RNA polymerase II transcription factor activity, and ‘sequence-specific DNA binding’ (GO:0000981, Table 2).

**Table 2:** GO analysis of differentially expressed genes

Category	Term	O	C	E	R	*P* Value
Up-regulated						
GOTERM_BP_DIRECT	GO:0051301~cell division	32	553	7.28	4.40	1.64E-12
	GO:0051276~chromosome organization	33	597	7.86	4.20	2.48E-12
	GO:0000819~sister chromatid segregation	19	215	2.83	6.71	6.47E-11
	GO:0007059~chromosome segregation	23	329	4.33	5.31	7.00E-11
	GO:0000278~mitotic cell cycle	40	972	12.79	3.13	9.53E-11
GOTERM_CC_DIRECT	GO:0005911~cell-cell junction	28	626	7.05	3.97	4.55E-10
	GO:0030054~cell junction	39	1357	15.28	2.55	4.45E-08
	GO:0000777~condensed chromosome kinetochore	10	101	1.14	8.79	2.05E-07
	GO:0000776~kinetochore	11	130	1.46	7.52	2.52E-07
	GO:0043296~apical junction complex	11	130	1.46	7.52	2.52E-07
GOTERM_MF_DIRECT	GO:0019899~enzyme binding	44	1756	22.14	1.99	6.09E-06
	GO:0098632~protein binding involved in cell-cell adhesion	14	288	3.63	3.86	1.79E-05
	GO:0098631~protein binding involved in cell adhesion	14	293	3.69	3.79	2.17E-05
	GO:0042802~identical protein binding	35	1330	16.77	2.09	2.39E-05
	GO:0098641~cadherin binding involved in cell-cell adhesion	13	277	3.49	3.72	5.15E-05
Down-regulated						
GOTERM_BP_DIRECT	GO:0009719~response to endogenous stimulus	62	1536	25.89	2.40	5.06E-11
	GO:0071495~cellular response to endogenous stimulus	53	1190	20.06	2.64	5.06E-11
	GO:0009725~response to hormone	42	832	14.02	3.00	1.65E-10
	GO:0032870~cellular response to hormone stimulus	34	608	10.25	3.32	8.17E-10
	GO:1901700~response to oxygen-containing compound	55	1445	24.35	2.26	6.68E-09
GOTERM_CC_DIRECT	GO:0005615~extracellular space	49	1385	17.58	2.79	3.25E-11
	GO:0005578~proteinaceous extracellular matrix	21	347	4.40	4.77	3.59E-09
	GO:0031012~extracellular matrix	25	503	6.39	3.92	6.13E-09
	GO:0042995~cell projection	42	1806	22.93	1.83	7.75E-05
	GO:0005925~focal adhesion	15	390	4.95	3.03	1.44E-04
GOTERM_MF_DIRECT	GO:0000981~RNA polymerase II transcription factor activity, sequence-specific DNA binding	31	652	10.49	2.95	6.92E-08
	GO:0003700~transcription factor activity, sequence-specific DNA binding	42	1203	19.36	2.17	1.53E-06
	GO:0001071~nucleic acid binding transcription factor activity	42	1204	19.38	2.17	1.56E-06
	GO:0000982~transcription factor activity, RNA polymerase II core promoter proximal region sequence-specific binding	19	342	5.50	3.45	2.96E-06
	GO:0001228~transcriptional activator activity, RNA polymerase II transcription regulatory region sequence-specific binding	17	323	5.20	3.27	2.00E-05

If there are more than five pathways in this category, the top five are selected according to the *P* value

*O* number of genes in this category in the user gene list, *E* expected number of genes in this category, *R* concentration ratio

The most enriched KEGG pathway term for the upregulated DEGs was ‘cell cycle’ (KEGG:04110) and for the downregulated DEGs was ‘complement and coagulation cascades’ (KEGG:04610, Table 3).

**Table 3:** KEGG enrichment analysis of differentially expressed genes

Term	C	O	E	R	*P* Value	Genes
Up-regulated						
hsa04110: Cell cycle	124	11	1.90	5.79	2.64E-06	E2F3, SFN, MCM4, BUB1, BUB1B, TTK, YWHAZ, CCNE1, CCNB2, CDK1, CDC20
hsa04530: Tight junction	139	9	2.13	4.22	0.00026240	CLDN4, CLDN3, CLDN7, TJP3, KRAS, LLGL2, PRKCI, CLDN10, MAGI1
hsa04114: Oocyte meiosis	124	8	1.90	4.21	0.00059620	ITPR3, BUB1, YWHAZ, CCNE1, CCNB2, CALML4, CDK1, CDC20
hsa01230: Biosynthesis of amino acids	75	6	1.15	5.22	0.00096638	PSAT1, IDH2, PFKP, PKM, PYCR1, TPI1
hsa00051: Fructose and mannose metabolism	33	4	0.51	7.91	0.00151933	PFKP, SORD, TPI1, TSTA3
Down-regulated						
hsa04610: Complement and coagulation cascades	79	7	1.42	4.93	0.00051053	PROCR, CFH, TFPI, THBD, C1S, C4BPB, C7
hsa04022: cGMP-PKG signaling pathway	168	10	3.02	3.31	0.00084616	AKT3, ADCY2, EDNRA, AKT2, GATA4, ITPR1, KCNJ8, MEF2C, PLN, RGS2
hsa04726: Serotonergic synapse	113	8	2.03	3.94	0.00092536	DUSP1, GNG4, GNG11, HTR2B, ITPR1, MAOA, MAOB, TRPC1
hsa04550: Signaling pathways regulating pluripotency of stem cells	142	9	2.55	3.53	0.00098921	AKT3, AKT2, APC, ID3, IL6ST, WNT4, TBX3, LEFTY2, KLF4
hsa04270: Vascular smooth muscle contraction	121	8	2.18	3.68	0.00144356	CALCRL, ADCY2, EDNRA, ITPR1, PPP1R12B, PLA2G1B, PLA2G5, CALD1

If there are more than five pathways in this category, the top five are selected according to the *P* value

*O* number of genes in this category in the user gene list, *E* expected number of genes in this category, *R* concentration ratio

### Hub gene and module screening from the PPI network

First, we determined the PPI network of the DEGs. The PPI network consisted of 275 nodes and 770 edges with a confidence score of more than 0.7 based on the STRING database. The top 12 hubs with degree centrality more than 29 were screened from the PPI network as hub genes. These hub genes included Cyclin-dependent kinase 1 (*CDK1*), DNA topoisomerase 2-alpha (*TOP2A*), cell-division cycle protein 2 (*CDC20*), G2/mitotic-specific cyclin-B2 (*CCNB2*), baculoviral inhibitor of apoptosis repeat-containing 5 (*BIRC5*), ubiquitin-conjugating enzyme E2 C2 (*UBE2C*), budding uninhibited by benzimidazoles 1 (*BUB1*), non-SMC condensin I complex subunit G (*NCAPG*), Ribonucleoside-diphosphate reductase subunit M2 (*RRM2*), Kinesin-like protein (*KIF2C*), centromere protein A (*CENPA*), and maternal embryonic leucine zipper kinase (*MELK*).

**Fig. 3:**
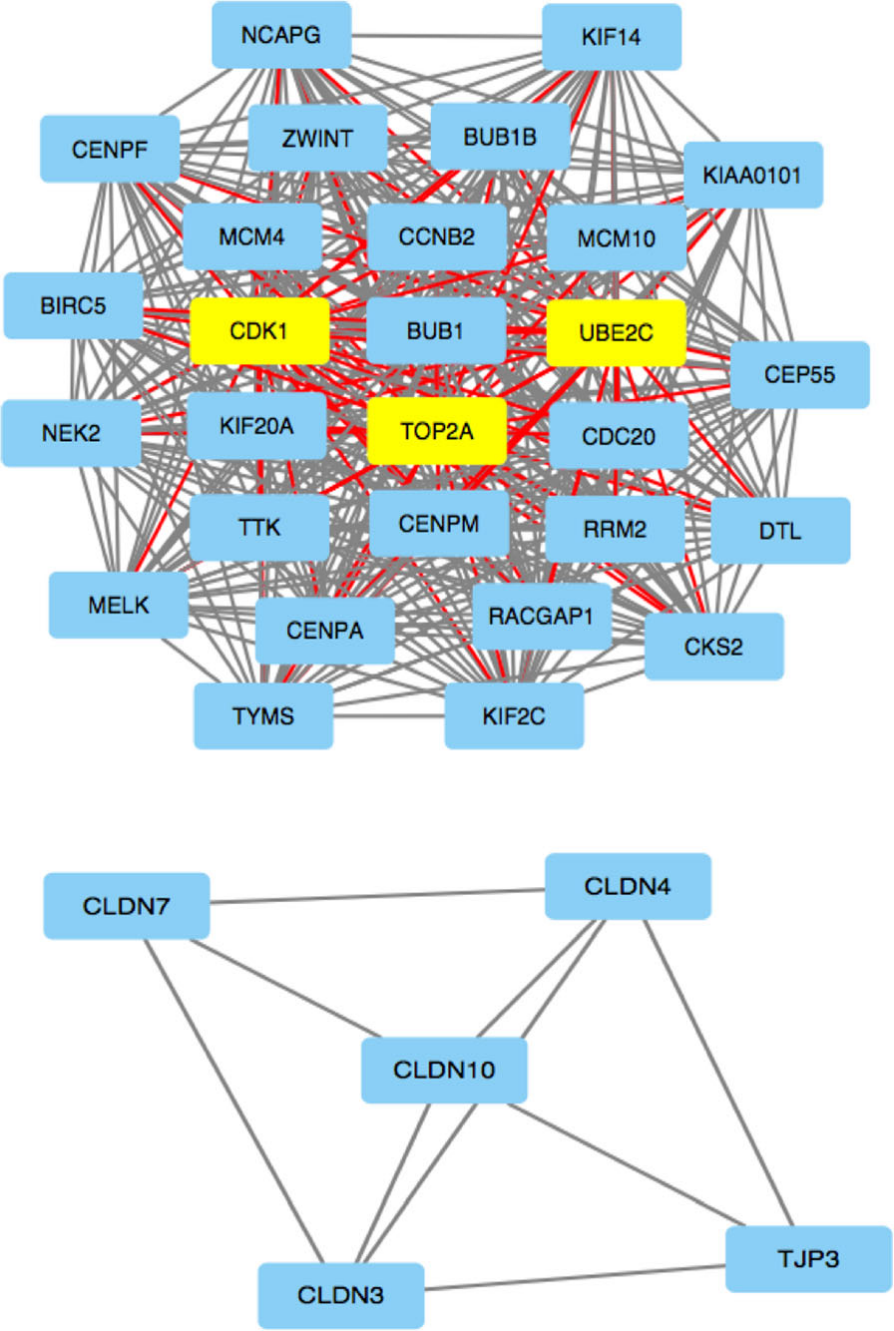
2 modules obtained from PPI network of DEGs using the MCODE software. The top 12 hub genes were all in the module 1

Moreover, the top 2 significant modules were obtained from the PPI network of DEGs using the MCODE software (Fig. 3). Then, KEGG pathway enrichment analyses of the genes in these two modules were performed using the WEB-based GEne SeT AnaLysis Toolkit (Additional file 3: Table S3). The results demonstrated that the genes in module 1 were mainly associated with cell cycle, oocyte meiosis and the p53 signaling pathway, while the genes in module 2 were primarily in tight junction proteins, leukocyte transendo thelial migration, hepatitis C, and cell adhesion molecules (CAMs). The top 12 hub genes belonged to module 1 and confirmed the critical pathways associated with ovarian cancer. In conclusion, these essential genes provide new ideas for the treatment of ovarian cancer.

### KM plots for hub genes

**Fig. 4:**
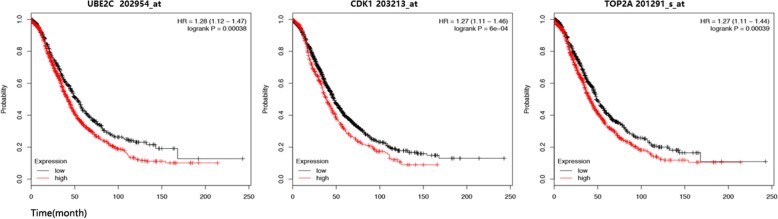
Top 3 genes (*CDK1*, *TOP2A* and *UBE2C*) significantly correlates with poor OS of ovarian cancer

The prognostic information of the 12 hub genes is freely available in http://kmplot.com/analysis/. The results demonstrated that the expression of *CDK1* (203213_at, HR 1.27 (1.11–1.46), *p* = 6 × 10^− 4^), *TOP2A* (201291_s_at, HR 1.27 (1.11–1.44), *p* = 3.9 × 10^− 4^), *CCNB2* (202705_at, HR 1.15 (1.22–1.81), *p* = 0.049), UBE2C (202954_at, HR 1.28 (1.12–1.47), *p* = 3.8 × 10^− 4^), *BUB1* (209642_at, HR 1.26 (1.08–1.46), *p* = 2.9 × 10^− 3^), *NCAPG* (218663_at, HR 1.26 (1.09–1.46), *p* = 1.9 × 10^− 3^), *RRM2* (201890_at, HR 1.17 (1.03–1.34), *p* = 1.7 × 10^− 2^), *KIF2C* (209408_at, HR 1.15 (1.01–1.32), *p* = 3.8 × 10^− 2^) and *CENPA* (204962_s_at, HR 1.23 (1.08–1.41), *p* = 2.4 × 10^− 3^) was negatively associated with the OS of epithelial ovarian cancer patients (Fig. 4 and Additional file 4: Figure S1).

## Discussion

The problematic diagnosis in an early stage and recurrence and resistance to current chemotherapeutic agents are the leading causes of high mortality in ovarian cancer based on data from The Surveillance, Epidemiology, and End Results (SEER) Program of the National Cancer Institute [21]. Therefore, the development of novel therapies for ovarian cancer is of great urgency. Previous research has proven that ovarian cancer is caused by the activation of oncogenes and the inactivation of cancer suppressor gene [22]. With continued advancements in high-throughput technologies, some genetic alterations associated with ovarian cancer, such as specific mutations in KRAS, loss-of-function mutations in PTEN, mutations in TP53, modifications in BRCA1/2, and changes in homologous recombination genes, have been uncovered [23]. Although a significant amount of data were produced by microarray studies, the sample sizes of most studies are small and may affect the identification of DEGs. However, meta-analysis of multiple microarray datasets makes the identification of DEGs more reliable by increasing the sample size.

In the present study, we performed a meta-analysis to determine the DEGs between ovarian cancer and normal ovarian tissues. We identified 563 DEGs, including 245 upregulated and 318 downregulated DEGs, in ovarian tissues by combining *p*-values (cutoff value of 5) and Z scores (cutoff value of 7). We classified the DEGs into functional categories based on their GO functions and KEGG pathways. Furthermore, we screened the following top 12 hub nodes with degree centrality more than 29 from the PPI network as hub genes: *CDK1, TOP2A, CDC20, CCNB2, BIRC5, UBE2C, BUB1, NCAPG, RRM2, KIF2C, CENPA*, and *MELK*. Additionally, we obtained two top significant modules from PPI networks of DEGs using MCODE analysis. Genes in module 1 were mainly associated with cell cycle, oocyte meiosis and the p53 signaling pathway, while genes in module 2 were primarily enriched in tight junction proteins, leukocyte transendothelial migration, hepatitis C, and CAMs.

Among the top 12 hub genes, nine hub genes were associated with poor OS in epithelial ovarian cancer patients. Based on GO functional analysis, KEGG pathway analysis, and survival analysis, we found that *CDK1*, *TOP2A*, and *UBE2C* might be the core genes contributing to the development of epithelial ovarian cancer at the molecular level.

CDK1 plays a vital role in the regulation of the cell cycle by modulating the centrosome. CDK1 not only promotes G2-M transition but also regulates G1 progression and G1-S transition by binding with multiple interphase cyclins [24, 25] In ovarian cancer, the expression of CDK1 is significantly associated with survival status, histological grade, FIGO stage, lymph node metastasis, and metastasis in epithelial ovarian cancer patients [26].

TOP2A is a nuclear enzyme involved involved in cell division and the cell cycle. TOP2A controls topological states of DNA by transiently breaking and subsequently rejoining of DNA strands [27]. Additionally, TOP2A is a direct molecular target of topoisomerase inhibitor, and its upregulation has been reported in several cancers including lung, nasopharyngeal, esophageal, gallbladder, hepatocellular, colorectal, breast, endometrial, pancreatic and ovarian cancer [28–31].

UBE2C, an essential factor of the anaphase-promoting complex/cyclosome (APC/C), is required for the destruction of mitotic cyclins and cell cycle progression [32]. The N-terminal extension of UBE2C contributes to the regulation of APC/C activity for substrate selection and checkpoint control [33]. UBE2C, the exclusive partner of APC/C, participates in the degradation of the APC/C target protein family by initiating the formation of a Lys11-linked ubiquitin chain. Thus, UBE2C plays a vital role in the destruction of mitotic cyclins and other mitosis-related substrates. During early mitosis, the APC is activated through cyclin B/Cdk1-dependent phosphorylation and binding of its activator CDC20. During metaphase, UBE2C degrades securin and cyclin B by APC/C^CDC20^ to promote progression to anaphase [34]. UBE2C is significantly upregulated in several types of cancer including bladder, breast, brain, cervical, esophageal, colorectal, liver, lung, nasopharyngeal, prostate (late-stage), pancreatic, thyroid, stomach, and ovarian cancer [33]. UBE2C is associated with tumor progression. I. van Ree et al. identified UBE2C as a prominent proto-oncogene that contributes to whole chromosome instability and tumor formation over a wide range of overexpression levels [35].

In our study, in addition to *UBE2C* upregulation, *CDC20, CDK1,* and *CCNB2* are overexpressed. Combined with the above results, it is logic to assume that the interaction among UBE2C, CDC20, CDK1, and CCNB2 may play a vital role in the formation and development of ovarian cancer. Overexpression of *UBE2C* was associated with poor OS for ovarian cancer patients, and thus UBE2C might be a promising prognostic molecular biomarker and therapeutic target for ovarian cancer. It is worth to emphasize that though a series of distinct molecular and histologic subtypes of ovarian cancer exists and each subtype has different tumor microenvironment. The present research mainly focuses on those common pathways of epithelial ovarian cancer. Yet we have analyzed the corresponding gene expression data acquired from GSE9891 and the results shows that 11 out of 12 hub genes (expression data of CDK1 cannot be found in the datasets GSE9891) are significantly up-regulated (Additional file 5: Figure S2, Additional file 6: Figure S3, Additional file 7: Figure S4, Additional file 8: Figure S5, Additional file 9: Figure S6, Additional file 10: Figure S7, Additional file 11: Figure S8, Additional file 12: Figure S9, Additional file 13: Figure S10, Additional file 14: Figure S11 and Additional file 15: S12, Additional file 16: Table S4) in all of the molecular subtypes (differential, immunoreacted, proliferation and mesenchymal) comparing to control group. Subsequent researches will be conducted to investigate the role each hub gene played in each subtype of ovarian cancer,

Besides, we consider the protein expression of these hub genes might be instructive to the further study. The protein expression data of hub genes is acquired from the Human Protein Atlas for evaluation. The protein expressions of 5 hub genes (CDK1, TOP2A, CDC20, NCAPG, and MELK) are significantly up-regulated in ovarian cancer compared to normal tissues (Additional file 17: Table S5.). Also, we have done a chi-square analysis to explore the relationship between the expression of 12 hub genes and the metastasis of ovarian cancer. The results show none of these genes has connections to the cancer metastasis (*P* > 0.05). Overall, the present study was designed to identify DEGs through integrated bioinformatics analysis to find potential biomarkers and predict the development and prognosis of ovarian cancer. However, to obtain more accurate correlation results, we need to performed a series of validation experiments. In conclusion, this study provides robust evidence for future genomic-based individualized treatment of ovarian cancer.

## Conclusion

Until now, a large-scale meta-analysis identifying DEGs was absent from the ovarian cancer literature. Our study systematically validated previous studies and filled the gap regarding a large-scale meta-analysis in the field of ovarian cancer. Moreover, our meta-analysis identified three specific genes, namely, *CDK1*, *TOP2A* and *UBE2C*, which may be potential targets of ovarian cancer. Thus, this study provides convincing evidence for future genomic individualized treatment of epithelial ovarian cancer.

## Additional files

Additional file 1: **Table S1.** Information of 245 upregulated DEGs. (XLSX 49 kb)

Additional file 2: **Table S2.** Information of 318 downregulated DEGs. (XLSX 59 kb)

Additional file 3: **Table S3.** KEGG pathway enrichment analyses of the genes in the two modules using the WEB-based GEne SeT AnaLysis Toolkit. (XLSX 13 kb)

Additional file 4: **Figure S1.** The Kaplan-Meier plots of 9 hub gene selected from PPI network are associated with poor prognosis of ovarian cancer patients. (TIF 893 kb)

Additional file 5: **Figure S2.** The difference of BIRC5 expression level in four molecular subtypes of epithelial ovarian cancer to control group. (PDF 5 kb)

Additional file 6: **Figure S3.** The difference of BUB1 expression level in four molecular subtypes of epithelial ovarian cancer to control group. (PDF 5 kb)

Additional file 7: **Figure S4.** The difference of CCNB2 expression level in four molecular subtypes of epithelial ovarian cancer to control group. (PDF 4 kb)

Additional file 8: **Figure S5.** The difference of CDC20 expression level in four molecular subtypes of epithelial ovarian cancer to control group. (PDF 5 kb)

Additional file 9: **Figure S6.** The difference of CENPA expression level in four molecular subtypes of epithelial ovarian cancer to control group. (PDF 5 kb)

Additional file 10: **Figure S7.** The difference of KIF2C expression level in four molecular subtypes of epithelial ovarian cancer to control group. (PDF 5 kb)

Additional file 11: **Figure S8.** The difference of MELK expression level in four molecular subtypes of epithelial ovarian cancer to control group. (PDF 4 kb)

Additional file 12: **Figure S9.** The difference of NCAPG expression level in four molecular subtypes of epithelial ovarian cancer to control group. (PDF 5 kb)

Additional file 13: **Figure S10.** The difference of RRM2 expression level in four molecular subtypes of epithelial ovarian cancer to control group. (PDF 5 kb)

Additional file 14: **Figure S11.** The difference of TOP2A expression level in four molecular subtypes of epithelial ovarian cancer to control group. (PDF 5 kb)

Additional file 15: **Figure S12.** The difference of UBE2C expression level in four molecular subtypes of epithelial ovarian cancer to control group. (PDF 5 kb)

Additional file 16: **Table S4.** The difference of 11 hub genes expression changes in four molecular subtypes of epithelial ovarian cancer to normal tissues. (XLSX 9 kb)

Additional file 17: **Table S5.** The protein expression changes of hub genes in the ovarian cancer. (XLSX 9 kb)

## Abbreviations

APC/C: Anaphase-promoting complex/cyclosome
BIRC5: Baculoviral inhibitor of apoptosis repeat-containing 5
BUB1: Budding uninhibited by benzimidazoles 1
CAMs: Cell adhesion molecules
CCNB2: G2/mitotic-specific cyclin-B2
CDC20: Cell-division cycle protein 2
CDK1: Cyclin-dependent kinase 1
CENPA: Centromere protein A
DEGs: Differentially expressed genes
EGA: The European Genome-phenome Archive
FDR: False-discovery rate
GEO: Gene Expression Omnibus
GO: Gene ontology
GPLs: The GEO Platform Files
HGSC: High-grade serous carcinoma
HRs: Hazard ratios
KEGG: Kyoto Encyclopedia of Genes and Genomes
KIF2C: Kinesin-like protein
KM plotter: Kaplan-Meier plotter
MCODE: Molecular Complex Detection
MELK: Maternal embryonic leucine zipper kinase
NCAPG: Non-SMC condensin I complex subunit G
NCBI: National Center for Biotechnology Information
OS: Overall survival
PARP: Poly-ADP-ribose polymerase
PPIs: Protein-protein interactions
PRISMA: Systematic Reviews and Meta-Analyses
RRM2: Ribonucleoside-diphosphate reductase subunit M2
SEER: The Surveillance, Epidemiology, and End Results
TCGA: The Cancer Genome Atlas
TOP2A: DNA topoisomerase 2-alpha
UBE2C: Ubiquitin-conjugating enzyme E2 C2
