# Supplementary figures and images for: Identification of core genes in ovarian cancer by an integrative meta-analysis
Source: J Ovarian Res. 2018 Nov 19;11:94. doi: 10.1186/s13048-018-0467-z (PMC6240943; doi:10.1186/s13048-018-0467-z)

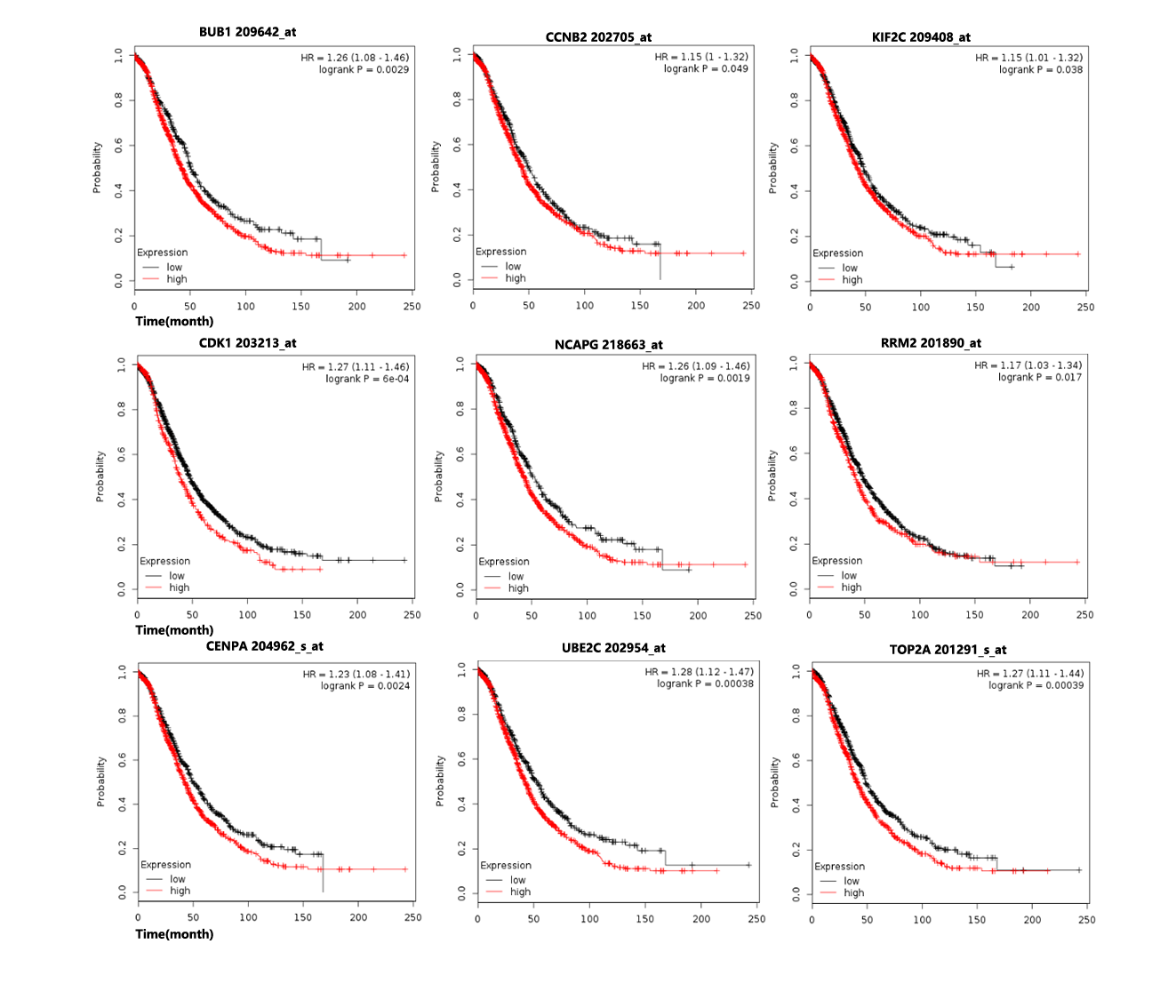

Supplement: Supplementary file 4 — Figure S1. The Kaplan-Meier plots of 9 hub gene selected from PPI network are associated with poor prognosis of ovarian cancer patients. (TIF 893 kb) [file 13048_2018_467_MOESM4_ESM.tif]

# BIRC5

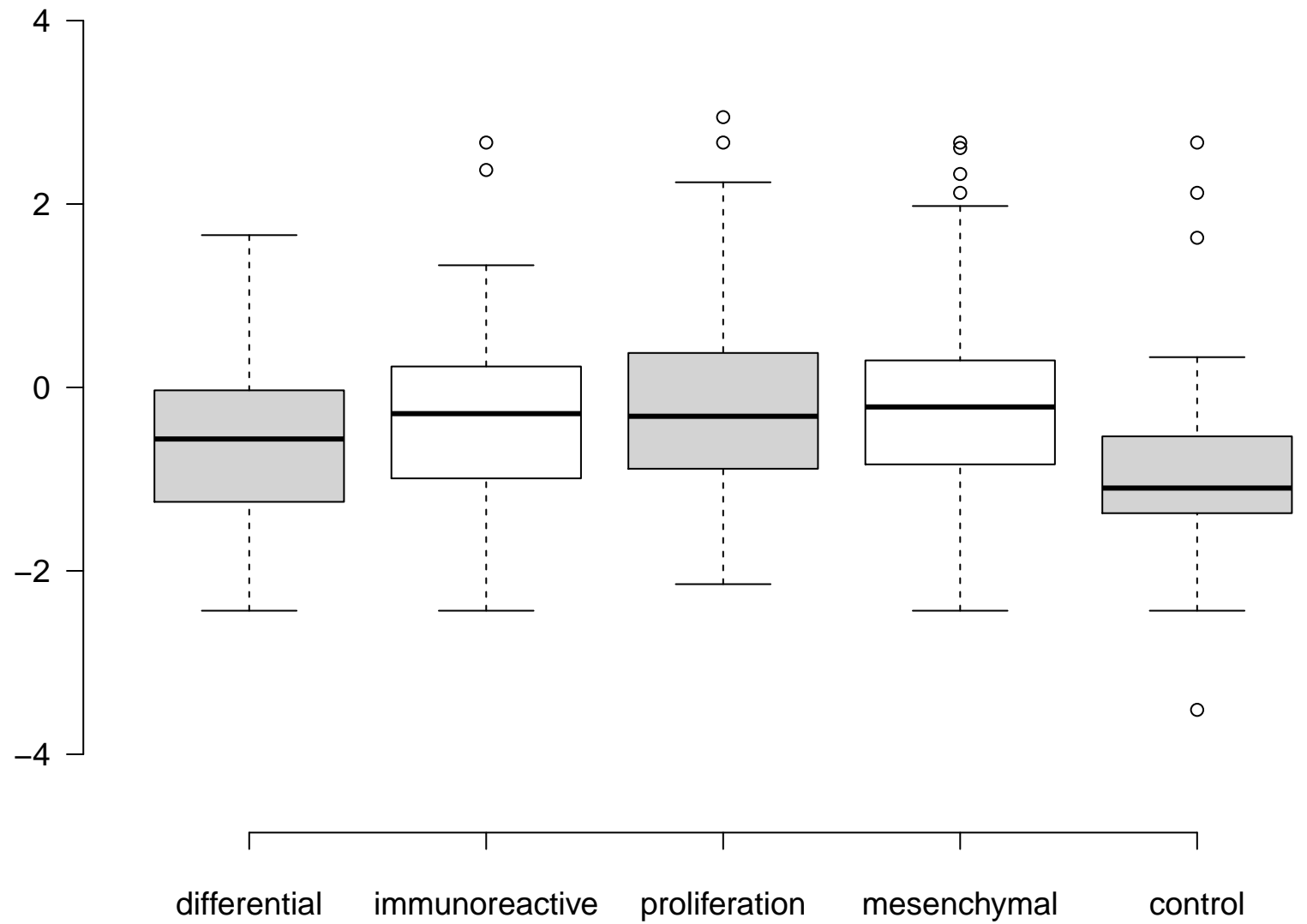

Supplement: Supplementary file 5 — Figure S2. The difference of BIRC5 expression level in four molecular subtypes of epithelial ovarian cancer to control group. (PDF 5 kb) [file 13048_2018_467_MOESM5_ESM.pdf]

# BUB1

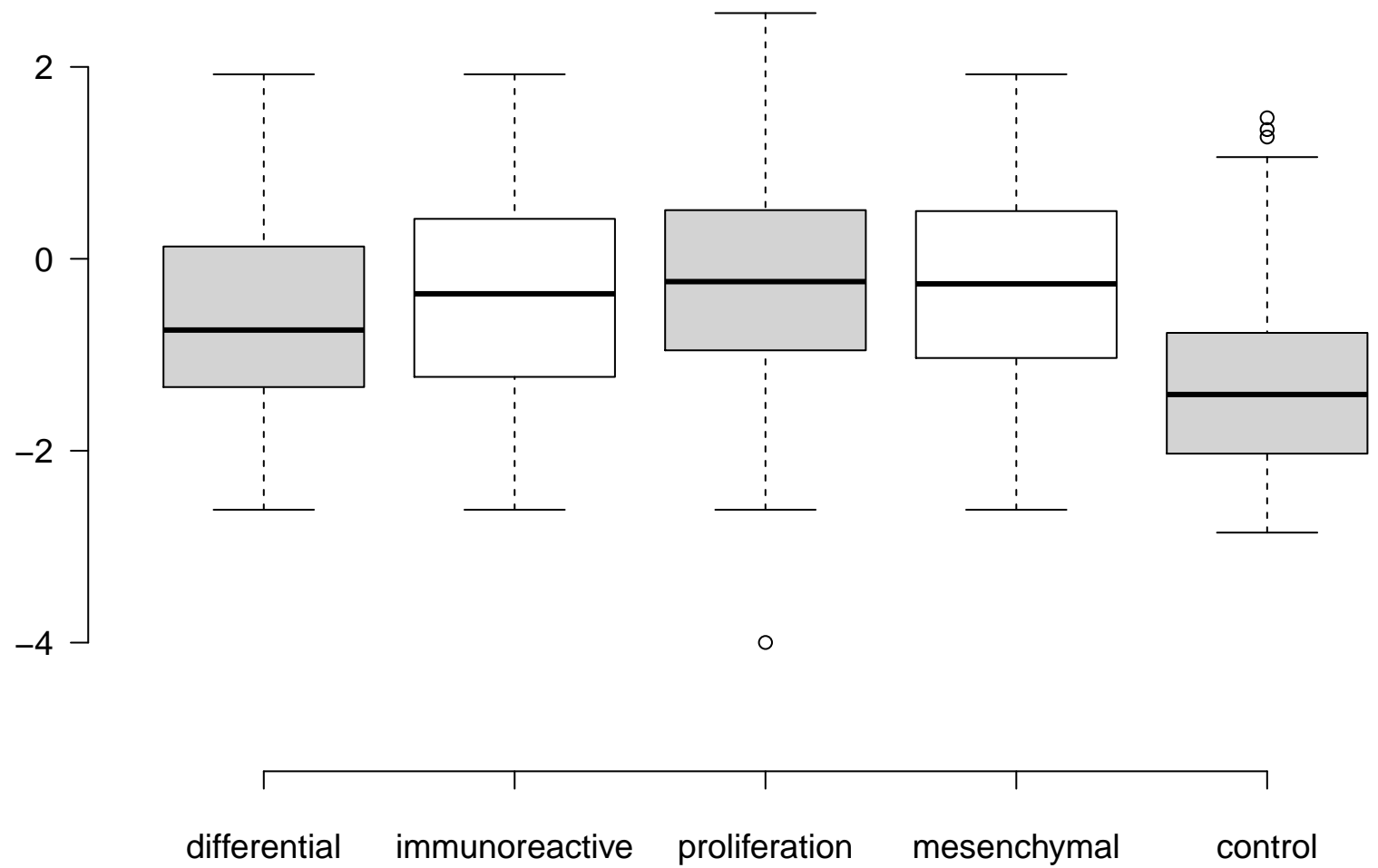

Supplement: Supplementary file 6 — Figure S3. The difference of BUB1 expression level in four molecular subtypes of epithelial ovarian cancer to control group. (PDF 5 kb) [file 13048_2018_467_MOESM6_ESM.pdf]

# CCNB2

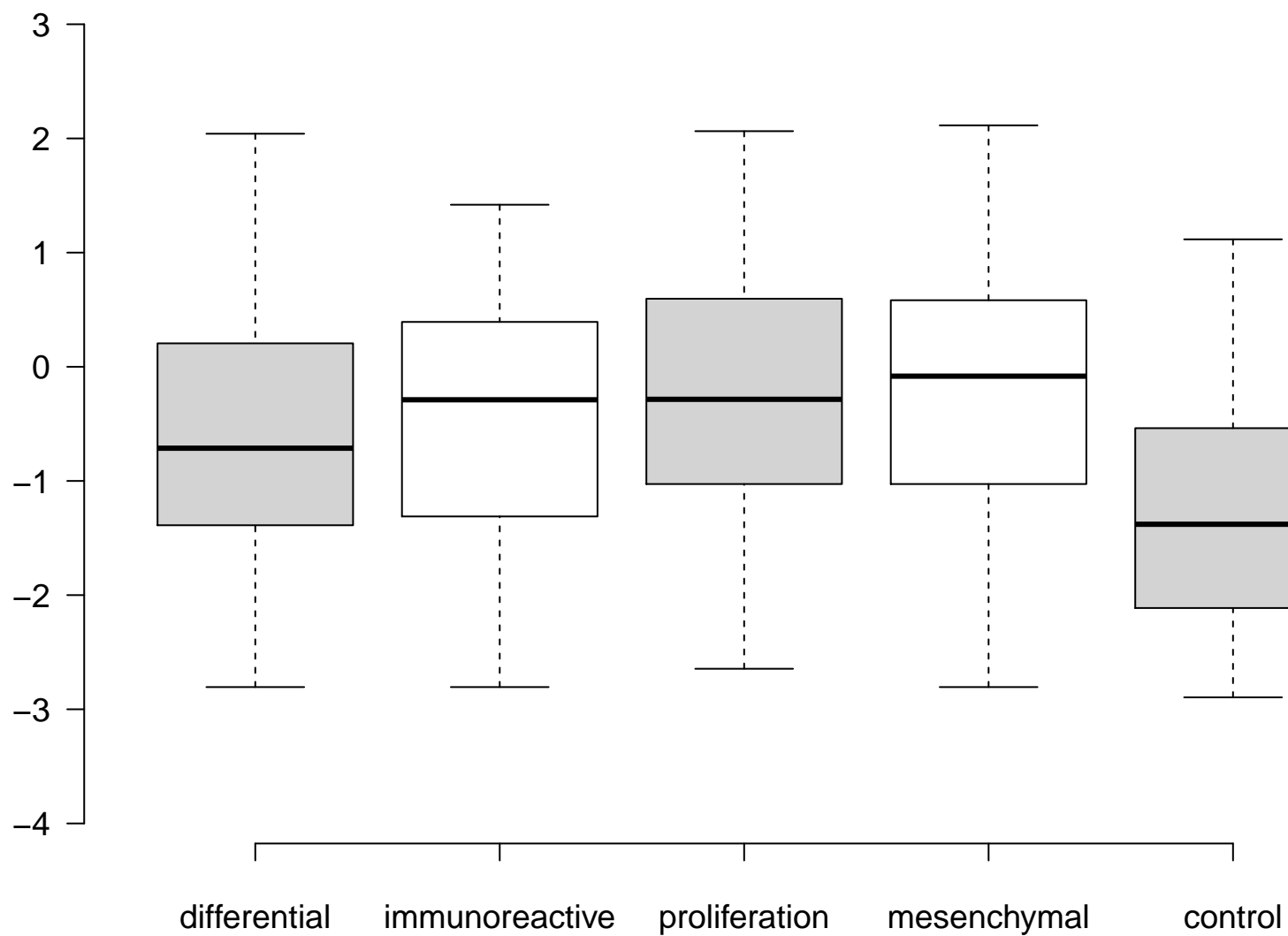

Supplement: Supplementary file 7 — Figure S4. The difference of CCNB2 expression level in four molecular subtypes of epithelial ovarian cancer to control group. (PDF 4 kb) [file 13048_2018_467_MOESM7_ESM.pdf]

# CDC20

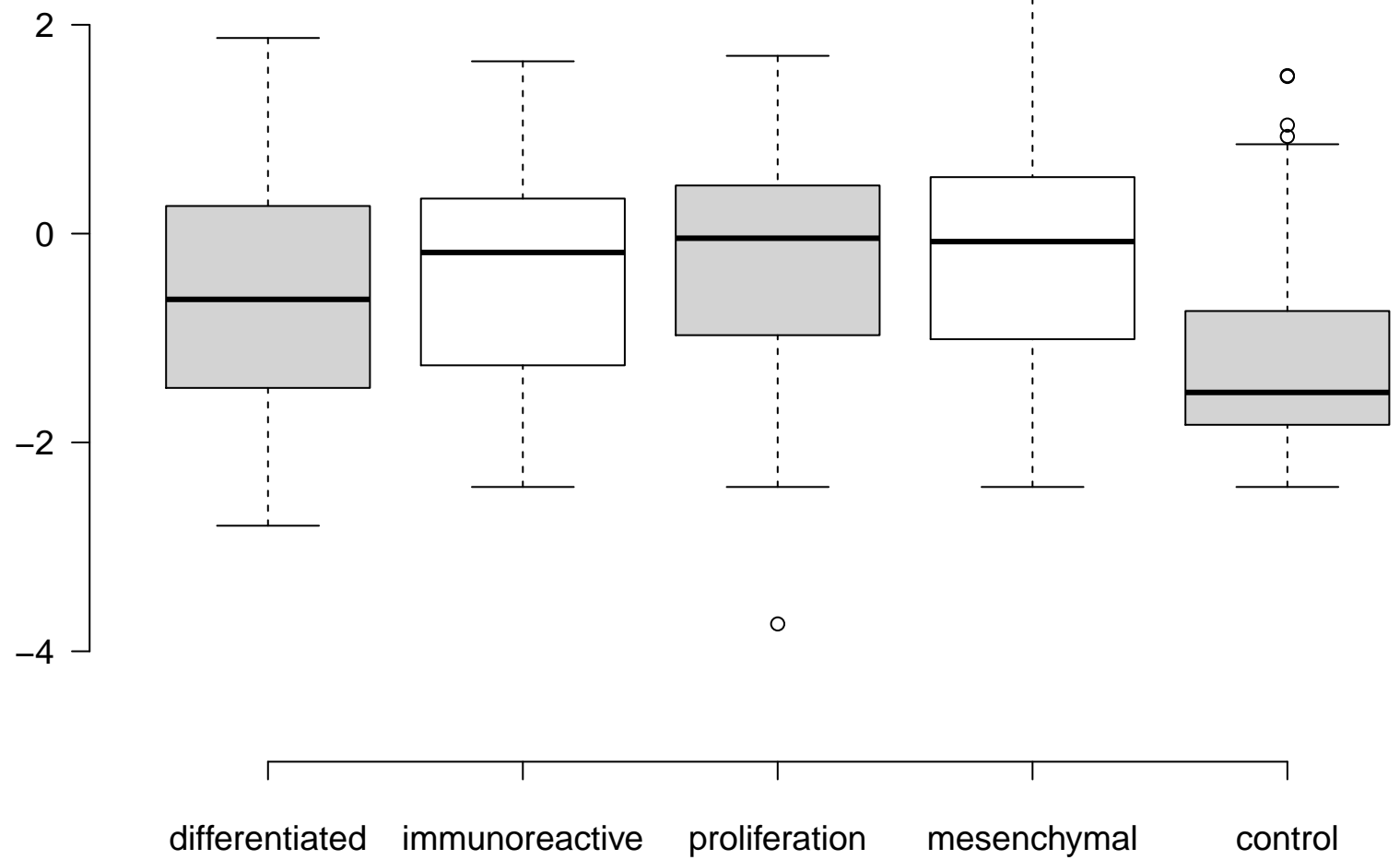

Supplement: Supplementary file 8 — Figure S5. The difference of CDC20 expression level in four molecular subtypes of epithelial ovarian cancer to control group. (PDF 5 kb) [file 13048_2018_467_MOESM8_ESM.pdf]

# CENPA

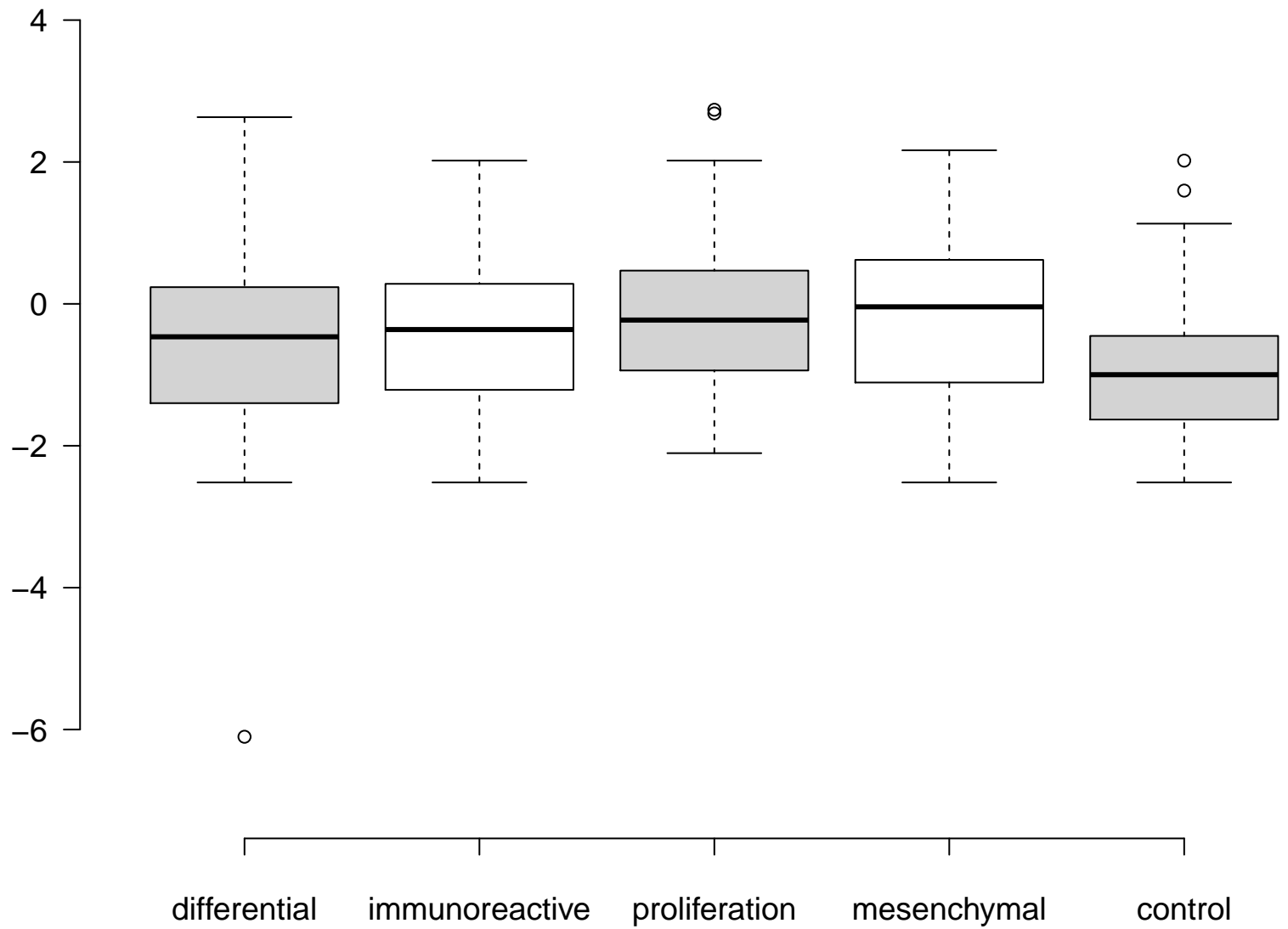

Supplement: Supplementary file 9 — Figure S6. The difference of CENPA expression level in four molecular subtypes of epithelial ovarian cancer to control group. (PDF 5 kb) [file 13048_2018_467_MOESM9_ESM.pdf]

# KIF2C

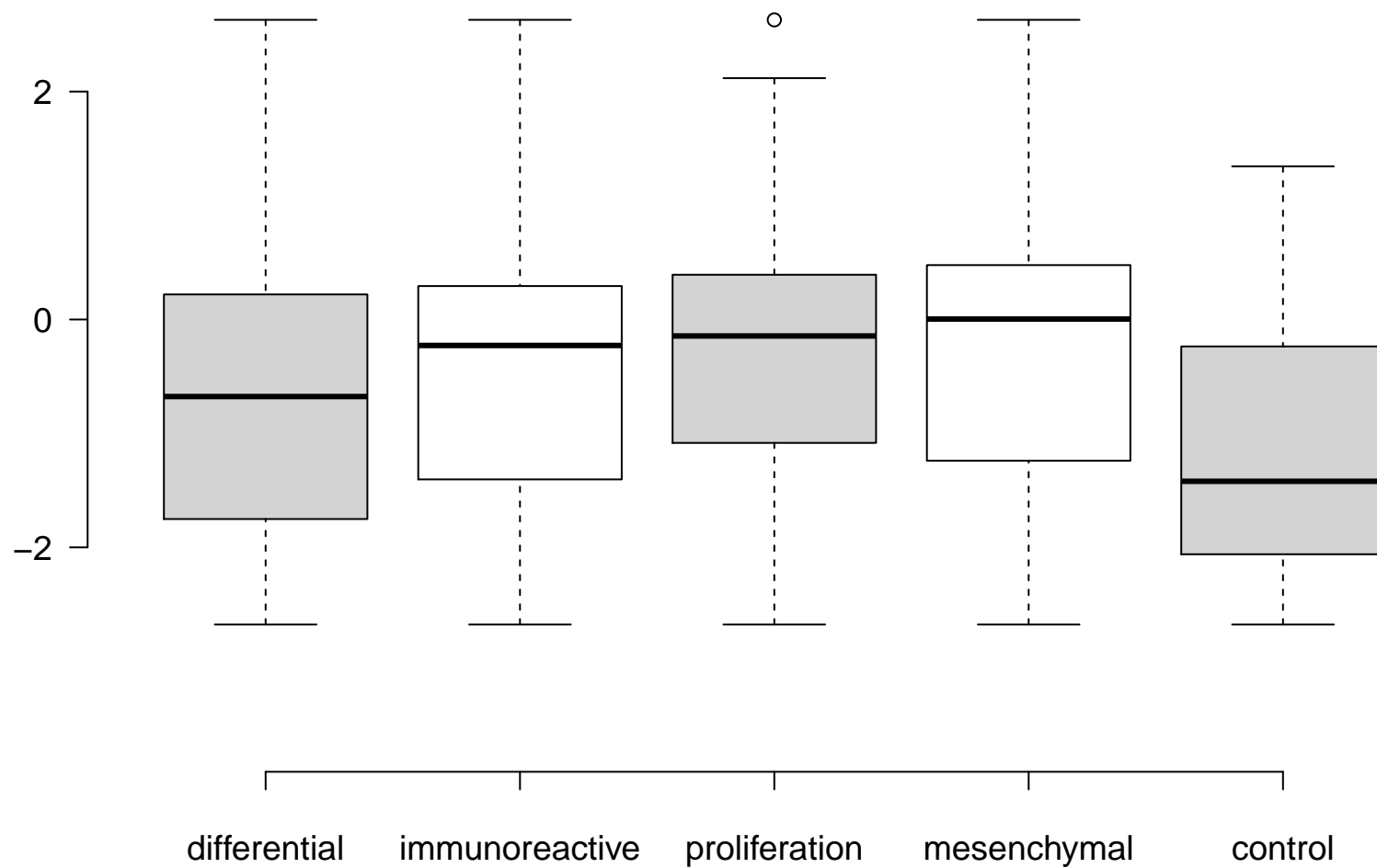

Supplement: Supplementary file 10 — Figure S7. The difference of KIF2C expression level in four molecular subtypes of epithelial ovarian cancer to control group. (PDF 5 kb) [file 13048_2018_467_MOESM10_ESM.pdf]

# MELK

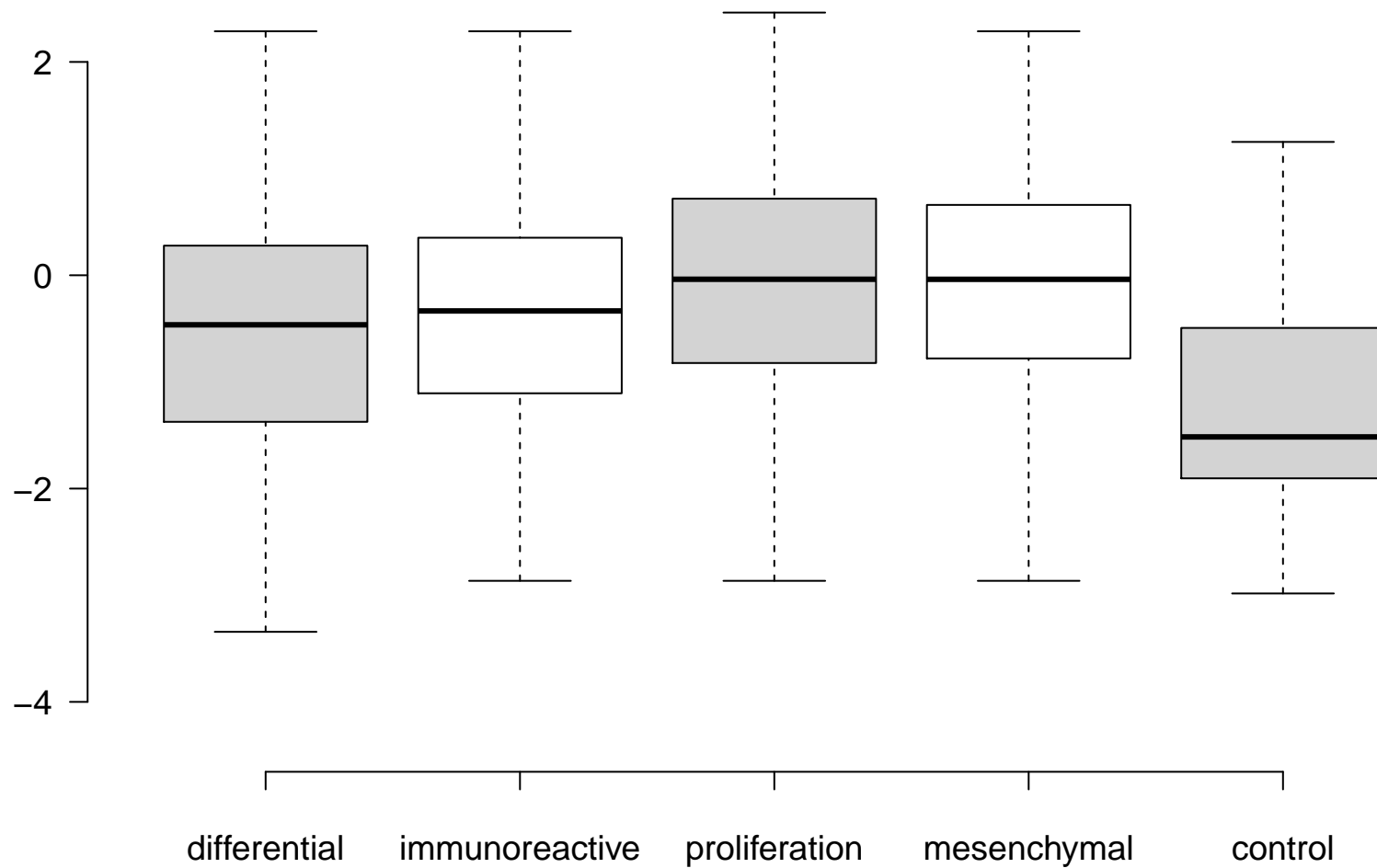

Supplement: Supplementary file 11 — Figure S8. The difference of MELK expression level in four molecular subtypes of epithelial ovarian cancer to control group. (PDF 4 kb) [file 13048_2018_467_MOESM11_ESM.pdf]

# NCAPG

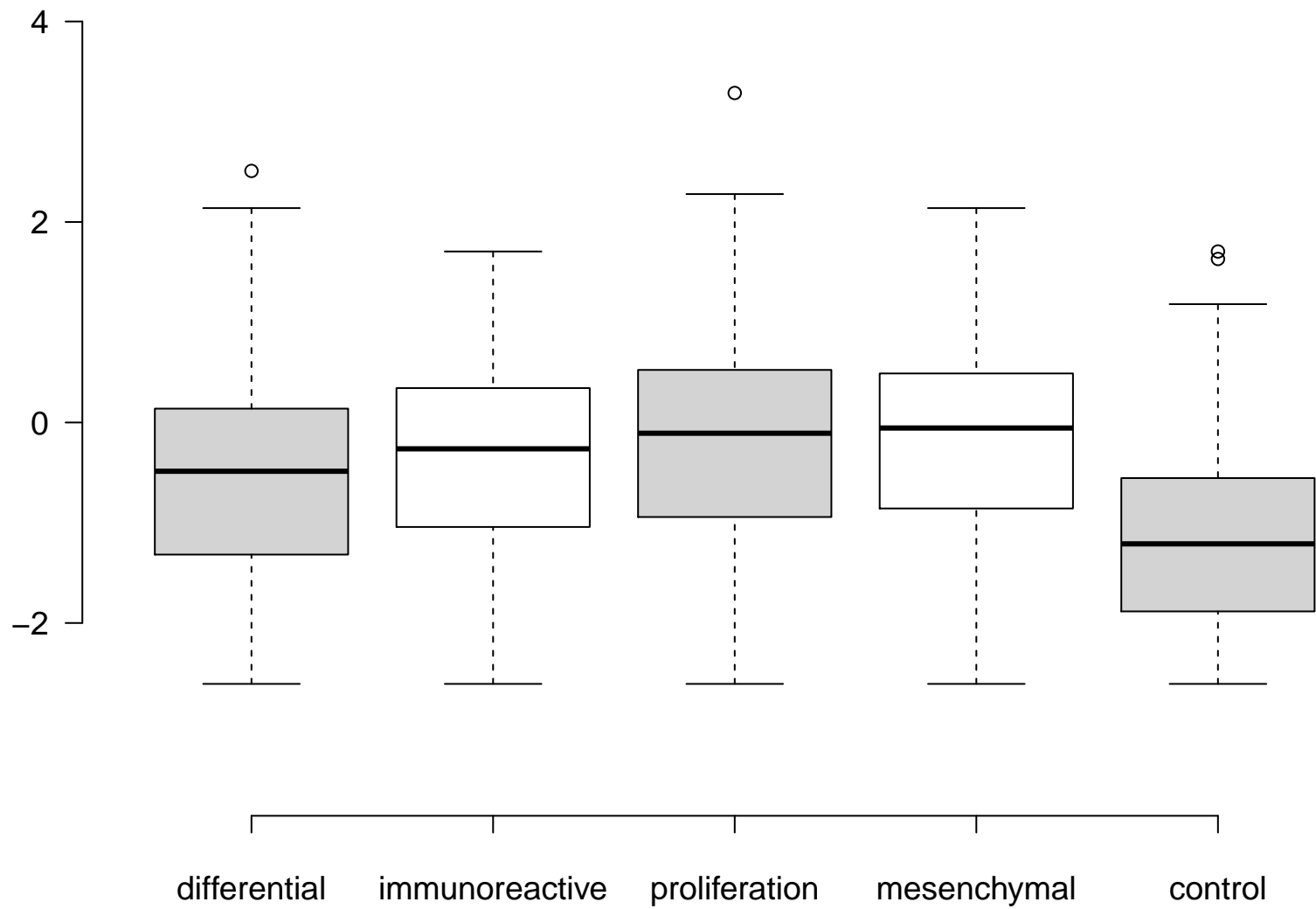

Supplement: Supplementary file 12 — Figure S9. The difference of NCAPG expression level in four molecular subtypes of epithelial ovarian cancer to control group. (PDF 5 kb) [file 13048_2018_467_MOESM12_ESM.pdf]

## RRM2

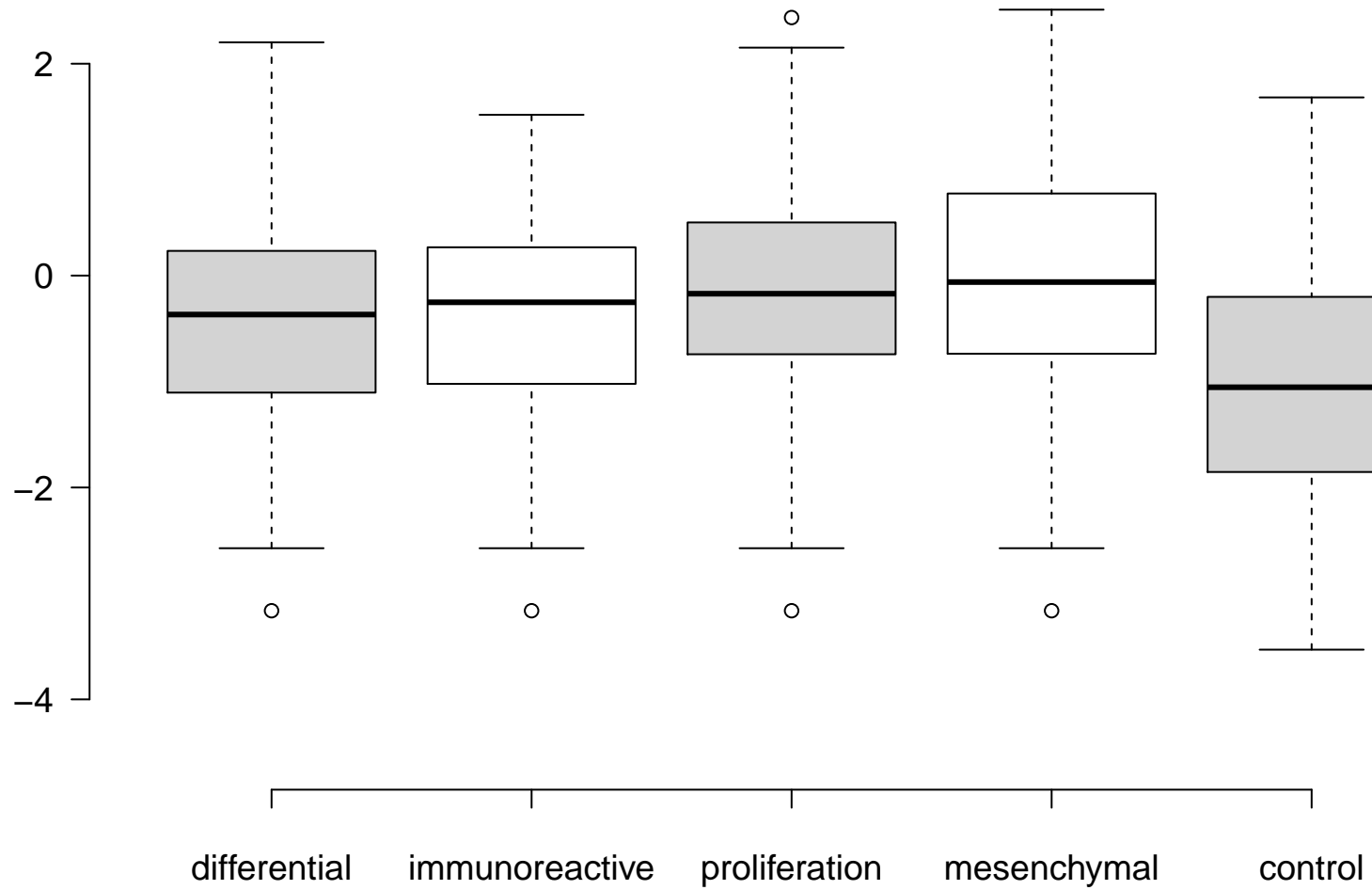

Supplement: Supplementary file 13 — Figure S10. The difference of RRM2 expression level in four molecular subtypes of epithelial ovarian cancer to control group. (PDF 5 kb) [file 13048_2018_467_MOESM13_ESM.pdf]

# TOP2A

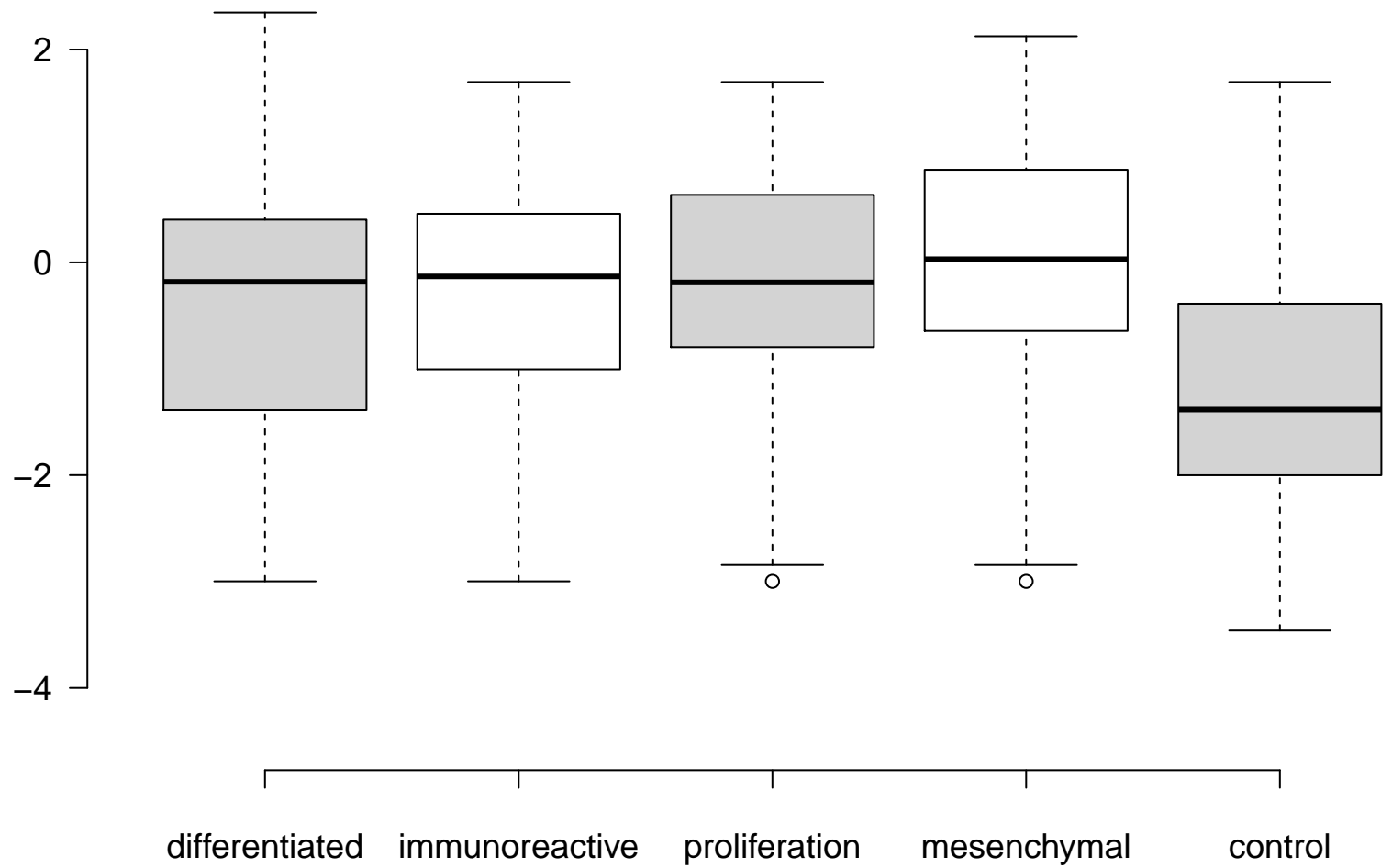

Supplement: Supplementary file 14 — Figure S11. The difference of TOP2A expression level in four molecular subtypes of epithelial ovarian cancer to control group. (PDF 5 kb) [file 13048_2018_467_MOESM14_ESM.pdf]

# UBE2C

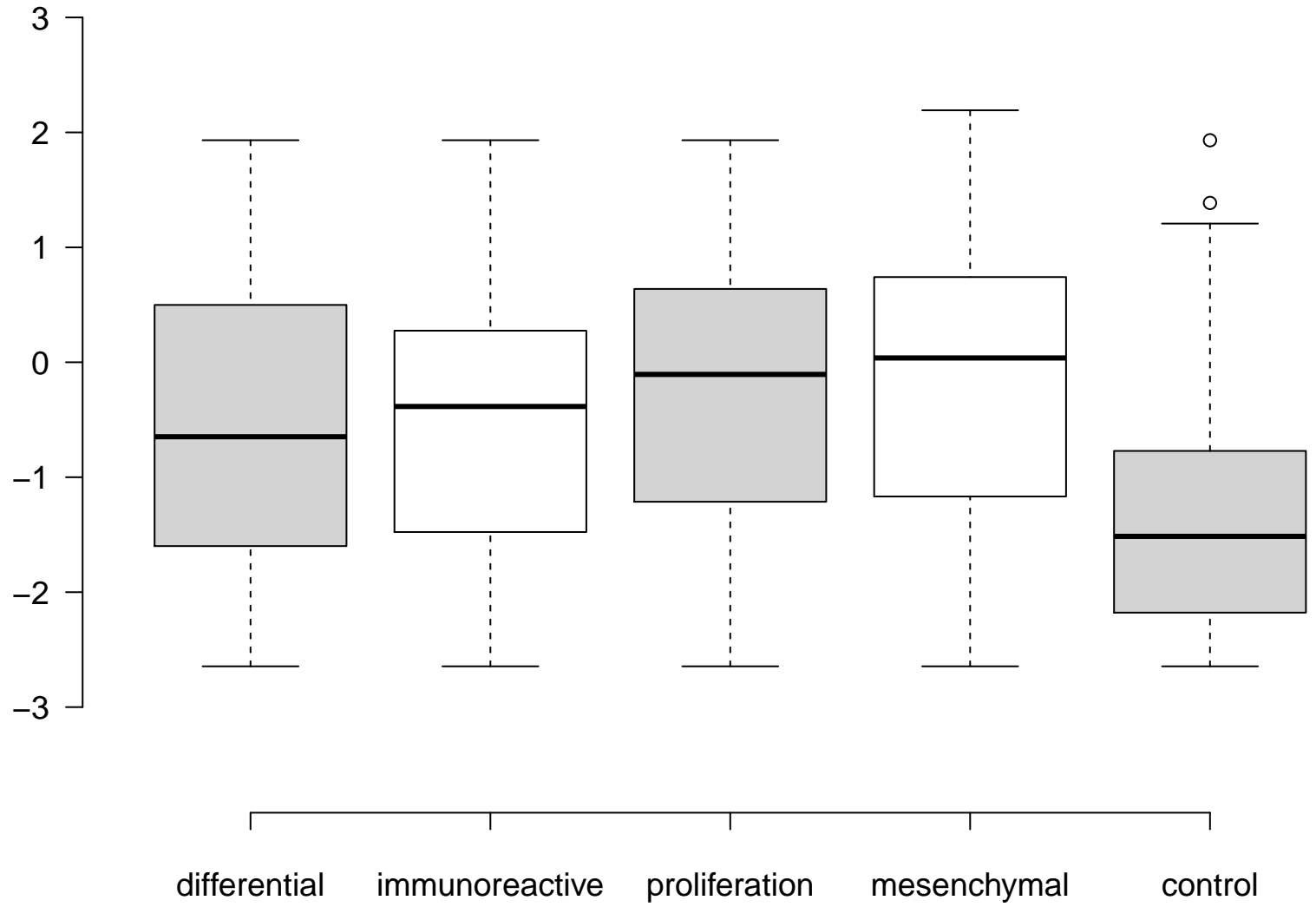

Supplement: Supplementary file 15 — Figure S12. The difference of UBE2C expression level in four molecular subtypes of epithelial ovarian cancer to control group. (PDF 5 kb) [file 13048_2018_467_MOESM15_ESM.pdf]
